# Inhibition of RIP3 increased ADSC viability under OGD and modified the competency of adipogenesis, angiogenesis, and inflammation regulation

**DOI:** 10.1042/BSR20212808

**Published:** 2022-03-29

**Authors:** Zhenyu Yang, Zuoliang Qi, Xiaonan Yang, Qiuni Gao, Yuling Hu, Xihang Yuan

**Affiliations:** Chinese Academy of Medical Sciences and Peking Union Medical College Plastic Surgery Hospital and Institute, Beijing, China

**Keywords:** adipogenesis, ADSC, angiogenesis, necroptosis, RIP3

## Abstract

Adipose-derived stem cells (ADSCs) showed decreased cell viability and increased cell death under oxygen-glucose deprivation (OGD). Meanwhile, vital necroptotic proteins, including receptor-interacting protein kinase (RIP) 3 (RIP3) and mixed lineage kinase domain-like pseudokinase (MLKL), were expressed in the early stage. The present study aims to explore the effect of necroptosis inhibition on ADSCs. ADSCs were obtained from normal human subcutaneous fat and verified by multidirectional differentiation and flow cytometry. By applying cell counting kit-8 (CCK-8), calcein/propidium iodide (PI) staining and immunostaining, we determined the OGD treatment time of 4 h, a timepoint when the cells showed a significant decrease in viability and increased protein expression of RIP3, phosphorylated RIP3 (pRIP3) and phosphorylated MLKL (pMLKL). After pretreatment with the inhibitor of RIP3, necroptotic protein expression decreased under OGD conditions, and cell necrosis decreased. Transwell assays proved that cell migration ability was retained. Furthermore, the expression of the adipogenic transcription factor peroxisome proliferator-activated receptor γ (PPARγ) and quantitative analysis of Oil Red O staining increased in the inhibitor group. The expression of vascular endothelial growth factor-A (VEGFA) and fibroblast growth factor 2 (FGF2) and the migration test suggest that OGD increases the secretion of vascular factors, promotes the migration of human umbilical vein endothelial cells (HUVECs), and forms unstable neovascularization. ELISA revealed that inhibition of RIP3 increased the secretion of the anti-inflammatory factor, interleukin (IL)-10 (IL-10) and reduced the expression of the proinflammatory factor IL-1β. Inhibition of RIP3 can reduce the death of ADSCs, retain their migration ability and adipogenic differentiation potential, reduce unstable neovascularization and inhibit the inflammatory response.

## Introduction

Autologous fat transplantation (AFT) has been widely used in recent years. This technique is favored in aesthetic and reconstructive surgeries because of its comprehensive source of adipose tissue, relatively simple access, and minimal invasion [1–3]. However, the sizeable long-term graft survival rate fluctuation and lack of corresponding intervention methods limit its further development and clinical application [4–7]. Based on the three-zone theory, the intermediate and central areas of the graft were under hypoxia and ischemia. It took approximately 1–3 months to promote angiogenesis and reconstruct the capillary network. Therefore, a lack of oxygen and glucose induces cell death of adipocytes and adipose-derived stem cells (ADSCs) at an early age [8].

In 2008, Yoshimura et al. were the first to clearly describe the cell-assisted lipotransfer (CAL) technique in AFT [9,10]. Since then, many articles have studied the use of stromal cells or mesenchymal stem cells in standard lipofilling and have found slight variations and promising results [11]. ADSCs were used due to their synergistic effects on survival and regeneration by paracrine secretion and recruitment effects, which may be inhibited under ischemic and hypoxic states.

Necroptosis is a proinflammatory lytic form of cell death with programmed properties occurring in numerous cells, including ADSCs. The signaling of this kind of programmed cell death is modulated by receptor-interacting protein kinase (RIP) 1 (RIP1), RIP3, and mixed lineage kinase domain-like pseudokinase (MLKL) when the activity of caspase-8 becomes compromised [12]. Following oxygen-glucose deprivation (OGD) treatment, the occurrence of necroptosis has been verified in hippocampal neurons, cardiomyocytes, endothelial cells, hepatocytes, and retinal ganglion cells. The benefit resulting from inhibiting RIP3 was observed in the models above [13–18].

We built an appropriate OGD *in vitro* culture model that can fit the environment faced by ADSCs *in vivo* and track its possible changes. In the present study, we hypothesized that ADSCs had a certain degree of necroptosis following OGD treatment, and the use of GSK′872, which is an inhibitor of RIP3, could improve adipogenic differentiation, angiogenesis, and inflammatory regulation to establish a theoretical basis for cell-assisted AFT in the future.

## Methods

### ADSCs isolation, cultivation, and identification

Human adipose tissue was obtained by abdominal liposuction of different female patients, washed twice with phosphate-buffered saline (PBS), and centrifuged at 200×***g*** for 5 min to remove impurities, including lower erythrocytes and upper oil. Rinsed samples were then mixed with the same volume of 0.25% (w/v) type 1 collagenase (Sigma–Aldrich, Germany), digested in a shaker at 125 rpm for 45 min, and centrifuged again. ADSCs in the centrifugation precipitate were resuspended in low-glucose Dulbecco’s modified Eagle’s medium (DMEM; Gibco; Thermo Fisher Scientific, U.S.A.), filtered through a 70-μm cell strainer, and seeded in a 10-cm culture dish. We cultured ADSCs with mesenchymal stem cell medium (MSCM 7501 ScienCell Research Laboratories, U.S.A.) at 37°C in 5% CO_2_ until they reached 80–90% confluence. Thereafter, they were dissociated with 1× TrypLE Express (Gibco; Thermo Fisher Scientific, U.S.A.) and passaged. The initial passage was defined as passage 0. Cells at passages 3–5 were used for all the experiments in the present study, including further characterization and* in vitro* differentiation. Informed consent was obtained from all donors. The following protocols and experiments were performed according to the Ethics Committee of Plastic Surgery Hospital, CAMS.

### ADSCs multilineage differentiation

#### Adipogenic differentiation assay

Human ADSCs were seeded into six-well plates precoated with 0.1% gelatin solution and then cultured in MSCM. When the cells reached 100% confluence, the medium was replaced with DMEM containing 10% FBS, 1% antibiotic/antimycotic solution, 0.5 mM isobutyl-methylxanthine, 1 μM dexamethasone, 10 μM insulin, and 200 μM indomethacin every 3 days and then replaced with DMEM containing 10% FBS, 1% antibiotic/antimycotic solution, and 10 μM insulin every alternate day (HUXMD-90031, Cyagen Bioscience, China) at 37°C under 5% CO_2_. After 21 days of culture, the cells were washed twice with PBS, fixed in 4% paraformaldehyde for 30 min, and stained with 0.3% Oil Red O solution for 30 min. After two washes with PBS, the cells were observed and photographed under a phase-contrast inverted microscope.

#### Osteogenic differentiation assay

Human ADSCs were seeded into six-well plates precoated with 0.1% gelatin solution and then cultured in DMEM containing 10% FBS, 1% antibiotic/antimycotic, 0.01 μM 1,25-dihydroxy vitamin D3, 50 μM ascorbate-2-phosphate, and 10 mM β-glycerophosphate (HUXMD-90021, Cyagen Bioscience, China). The medium was changed every 3 days. After 21 days of culture at 37°C under 5% CO_2_, the cells were washed twice with PBS, fixed in 4% paraformaldehyde for 30 min, and stained with 0.3% Alizarin Red for 5 min. After two washes with PBS, the cells were observed and photographed under a phase-contrast inverted microscope.

#### Chondrogenic differentiation assay

Human ADSCs (5 × 10^5^) were added to a 15-ml tube, washed twice with DMEM, and centrifuged at 200×***g*** for 5 min. The deposit was suspended in DMEM containing 10% FBS, 1% antibiotic/antimycotic, 10% dexamethasone, 1% transforming growth factor-β 3, and 1% ITS supplement (HUXMD-90041, Cyagen Bioscience, China), and the medium was replaced every 2–3 days. After 28 days of culture at 37°C under 5% CO_2_, the formed cartilage balls were fixed in formalin, embedded in paraffin wax, sectioned on to slides, dewaxed and dewatered, and stained with Alcian Blue for 30 min. After two washes with PBS, the sections were observed and photographed under a phase-contrast inverted microscope.

### Flow cytometry

Flow cytometry analysis was performed based on a published joint statement. Briefly, human ADSCs were detached, washed, and resuspended at a concentration of 1 × 10^7^ cells/ml. Antibodies against CD90, CD73, CD105, CD34, CD11b, CD19, and CD45 (562245, BD Biosciences, U.S.A.) [19] were added to the cell suspension. After 30 min of incubation in the dark, the cells were washed twice and loaded on a flow cytometer (BD Biosciences, U.S.A.).

Annexin V/PI double staining was performed to analyze the state of the cells pretreated with or without GSK′872 following OGD treatment. Briefly, three groups of cells were harvested, centrifuged, and resuspended in PBS at a concentration of 5 × 10^5^/ml. The cell suspension was incubated with annexin V conjugated with FITC and PI (556570, BD Biosciences, U.S.A.) for 20 min in the dark and loaded on a flow cytometer.

### Oxygen and glucose deprivation model and treatments

To imitate the environment *in vivo*, we cultured ADSCs under hypoxic and glucose-free conditions. Briefly, we replaced the medium of ADSCs from MSCM with Glu-free DMEM (Gibco; Thermo Fisher Scientific, U.S.A.) after a three washes with PBS. Then, the dishes were placed in an airtight chamber equipped with a vacuum air pump and an inflator (CelCulture Esco Micro Pte. Ltd., Singapore) and flushed by a gas mixture of 1% O_2_, 5% CO_2_, and 94% N_2_. Cells without inhibitor treatment were used as a control.

The cells were divided into three groups: (i) NC: normal control group cells were maintained in normal culture medium; (ii) OGD: normal control group cells with OGD treatment; (iii) GSK: cells pretreated with GSK′872 (1 μM, HY-101872 MedChemExpress LLC, U.S.A.) for 12 h before OGD treatment.

### Immunofluorescence studies

Cells seeded on climbing slides were washed twice with PBS, fixed in 4% paraformaldehyde, permeabilized with PBS containing 1% Triton X-100, blocked for 30 min, and then incubated with primary antibodies against RIP3 (1:100; ab62344, Abcam), phosphorylated RIP3 (pRIP3; 1:200; 93654, CST), and phosphorylated MLKL (pMLKL; 1:200; 91689, CST) overnight at 4°C. Anti-rabbit secondary conjugated with Alexa Fluor 488 (1:200; ab150117; Abcam) and α-tubulin conjugated with Alexa Fluor 555 (1:100; C1050, Beyotime, China) were simultaneously added to slides and incubated for 1 h at room temperature in the dark. After being washed with PBS, sections were mounted by mounting medium with 4′,6-diamidino-2-phenylindole (DAPI) (ab104139, Abcam). Fluorescent images were captured using fluorescence microscopy (Nikon).

### Proliferation assays

#### Calcein/PI staining

To evaluate the live/dead proportion of ADSCs after OGD treatment, we used a Calcein/PI Cell Viability/Cytotoxicity Assay Kit (C2015S, Beyotime, China) to double stain the cells at continuous time points during OGD treatment. Calcein acetoxymethyl ester could be hydrolyzed by esterase contained in live cells and emit green fluorescence, while propidium iodide could only penetrate the membrane of dead cells. After incubation, the cells were placed under a fluorescence microscope (Nikon), and the images were collected and semiquantitatively analyzed by ImageJ (version 1.53c).

#### Cell counting kit-8

The ability of the OGD model to induce ADSCs proliferation was assessed using an Enhanced Cell Counting Kit-8 (CCK-8; C0041, Beyotime, China) according to the manufacturer’s protocols. Briefly, 5000 cultured cells were seeded in 96-well plates overnight and then cultured in glucose-free DMEM under 1% O_2_ or in MSCM in a normal incubator as a control. Every 2 h, 10 μl of CCK-8 reagent was added to each well and incubated at 37°C for 2 h. The absorbance of each sample, which was proportional to the number of viable cells, was measured at a wavelength of 450 nm using a microplate reader. Each group was prepared in triplicates. The experiment was repeated three times.

To reduce the off-target effects, we used two inhibitors of RIP3, GSK′872 and GSK′843, to down-regulate necroptosis levels in cells. To verify the most appropriate concentration for ADSCs, we treated ADSCs with GSK′872 for 12 h from 0.25 to 10.0 µM (GSK′843 from 1.0 to 10.0 µM) compared with a control group with the same volume of DMSO in the medium and conducted CCK-8 assay for 3 days.

### Transwell assay

#### Coculture of ADSC supernatants and human umbilical vein endothelial cells

Conditioned medium from ADSCs was filtered through 0.22-μm filters and placed into the bottom of a 24-well plate, and then a supplement of 10% FBS was added to two OGD-treated groups. Human umbilical vein endothelial cells (HUVECs) were seeded into the 8-μm upper chamber (Falcon, Corning, U.S.A.) filled with 200 µl DMEM. After 12 h, the upper chambers were washed twice with PBS, fixed in 4% paraformaldehyde, permeabilized with methanol, stained with 0.1% Crystal Violet solution, and observed and photographed under a phase-contrast inverted microscope.

#### Cell migration

ADSCs were detached, harvested, and resuspended in 200 μl DMEM at a concentration of 2.5 × 10^5^/ml in the upper chamber with 500 μl DMEM containing 10% FBS added to the bottom of a 24-well plate. Subsequent procedures were described above.

### Western blotting analysis

For total protein extraction, the cells were washed with PBS, lysed in lysis buffer (87787, Thermo Fisher Scientific, U.S.A.) following the addition of a protease inhibitor, incubated on ice for 5 min, and centrifuged at 13000× ***g*** for 10 min at 4°C. Protein concentration was determined with a bicinchoninic acid assay (BCA) kit (P0012, Beyotime). We separated proteins using 10% SDS/PAGE and transferred them to PVDF membranes. The membranes were blocked with 5% nonfat milk for 1 h at room temperature and incubated with primary antibodies against RIP3 (1:1000; ab62344, Abcam), pRIP3 (1:1000; 93654, CST), MLKL (1:1000; ab184718, Abcam), pMLKL (1:1000; 91689, CST), peroxisome proliferator-activated receptor γ (PPARγ; 1:1000; ab178860, Abcam), vascular endothelial growth factor-A (VEGFA; 1:1000; 19003-1-AP, Proteintech), and glyceraldehyde 3-phosphate dehydrogenase (GAPDH; 1:50000; 60004-1, Proteintech) as an internal control at 4°C overnight. After being extensively washed with Tris-buffered saline with Tween 20 (TBST), the membranes were incubated with anti-rabbit/mouse secondary antibodies (1:10000; SA00001-1/2, Proteintech) for 1 h at room temperature. Protein was developed with ECL reagent (Millipore; Sigma–Aldrich, Germany) and visualized using a ChemiDoc MP Imager (Bio-Rad Laboratories, U.S.A.). The density of bands was determined by ImageJ (version 1.53c).

### Enzyme-linked immunosorbent assay

Quantification of secreted proteins in cell supernatants was performed using ELISA. Conditioned medium of different groups of ADSCs was harvested, centrifuged, and assessed for inflammatory cytokines using commercially available ELISA kits for interleukin (IL)-1β (IL-1β; JL13662, J&L Biological, China) and IL-10 (JL19246, J&L Biological, China) according to the manufacturer’s instructions. The concentration of each paracrine factor was calculated in pg/ml, and each group was prepared in triplicate.

### Quantitative reverse transcription polymerase chain reaction

Total RNA was isolated from the control, OGD-treated, and OGD-treated groups after GSK′872 pretreatment using the TRIzol method. RNA purity was evaluated by calculating the A260/A280 ratio, which should be between 1.8 and 2.0. cDNA was reverse transcribed from isolated RNA (1 μg) using a cDNA synthesis kit (AT311, TransGen Biotech, China). Subsequently, real-time quantitative polymerase chain reaction (qPCR) was performed using cDNA as a template and KAPA SYBR FAST qPCR Master Mix (2×) Universal (KK4601, Roche; Sigma–Aldrich, Germany) according to the manufacturer’s instructions. Primers were synthesized by Tsingke Biotechnology Co. (Beijing, China). Because of the slight variation in GAPDH amplification plots of ADSCs under a wide range of experimental settings, we chose GAPDH as the reference gene [20]. Target gene expression levels were run in triplicate, normalized to GAPDH, and quantified using the comparative *C*_t_ method to determine the gene expression fold change. The primers used for qRT-PCR analysis are listed in Table 1.

**Table 1:** Oligodeoxynucleotide primers used for qPCR

Gene	Primer sequence (5′→3′)	Product size (bp)
*PPARγ*	F: TGACTTCTCCAGCATTTCTACT	127
	R: AGGCTCCACTTTGATTGC	
*IL1β*	F: CCGACCACCACTACAGCAAG	260
	R: TGGACCAGACATCACCAAGC	
*IL10*	F: GGCGCTGTCATCGATTTCTTC	189
	R: ATAGAGTCGCCACCCTGATG	
*VEGFA*	F: CATGCAGATTATGCGGATCAA	82
	R: GCATTCACATTTGTTGTGCTGTAG	
*FGF2*	F: AAGAGCGACCCTCACATCAAG	227
	R: GTTCGTTTCAGTGCCACATACC	
*HGF*	F: GCCTCTGGTTCCCCTTCAATAG	117
	R: TGCGTCCTTTACCAATGATGC	
*GAPDH*	F: GGGAAACTGTGGCGTGAT	299
	R: GAGTGGGTGTCGCTGTTGA	

### Statistical analysis

All quantitative results are presented as the means ± standard error of the mean (SEM). Statistical comparisons were performed using one-way ANOVA in three independent experiments. GraphPad Prism version 9.0 software (GraphPad Inc., USA) was used for data analysis. Statistical significance was set at p<0.05.

## Results

### ADSCs displayed multipotent differentiation and expressed stem cell markers

**Figure 1:**
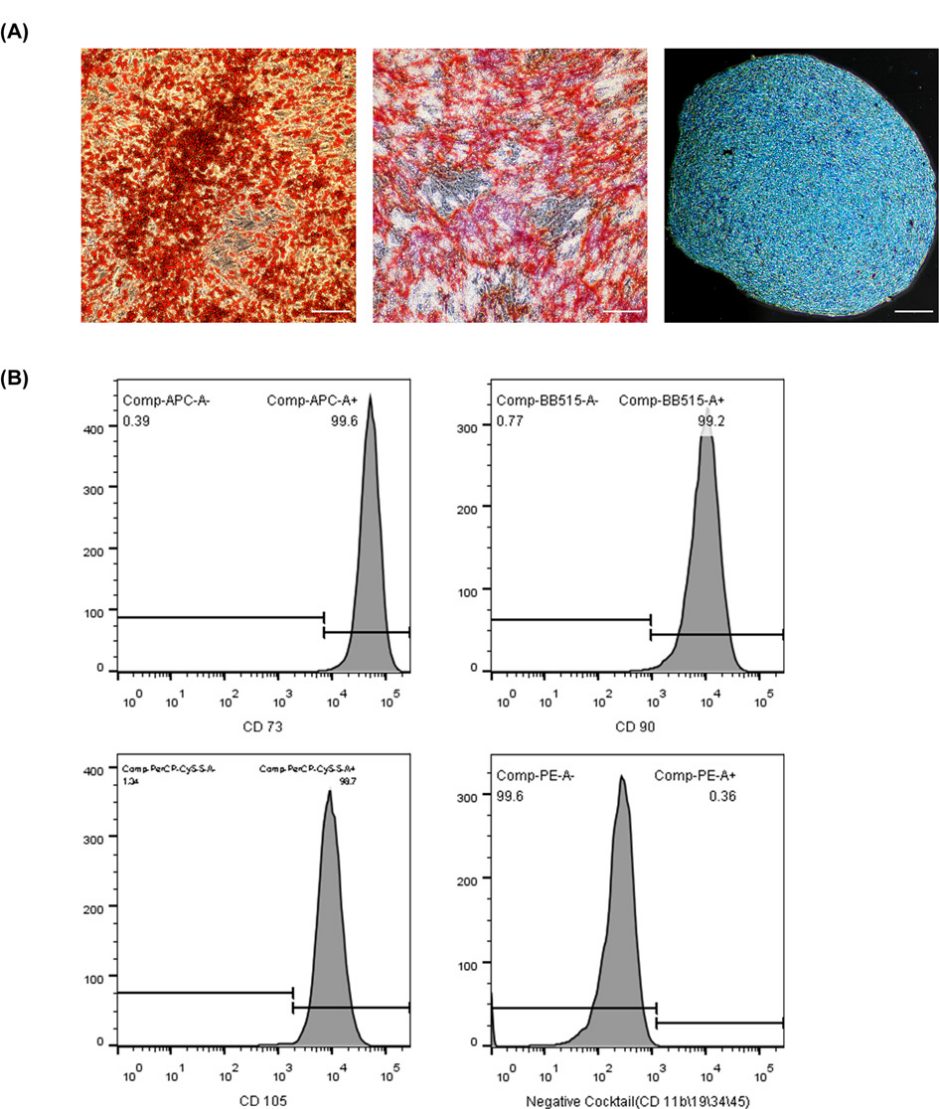
ADSC multilineage differentiation and identification of specific markers (**A**) Cells were induced to differentiate into adipocytes (left panel), osteoblasts (middle panel), and chondrocytes (right panel). Scale bars = 100 µm. (**B**) Flow cytometric characterization of ADSCs. ADSCs strongly expressed CD73 (99.6%), CD90 (99.2%), and CD105 (98.7%) and did not express CD11b, CD19, CD34, or CD45 (0.36%).

The multipotency of ADSCs was examined by osteogenic, chondrogenic, and adipogenic differentiation assays. ADSCs were induced with adipogenic medium, osteogenic medium, and chondrogenic medium for 3, 3, and 4 weeks, respectively. The staining image of phenotypes of adipogenesis, osteogenesis, and chondrogenesis showed the presence of lipid droplets in the cells stained with Oil Red O, calcium deposits stained with Alizarin Red, and cartilage stained with Alcian Blue (Figure 1A). The markers for phenotypical identification tested positive for CD73, CD90, and CD105 but negative for CD34, CD11b, CD19, and CD45 (Figure 1B). These results revealed that ADSCs isolated from human adipose tissue demonstrated typical ADSC characteristics.

### Cell proliferation and necroptosis occurrence following OGD treatment

**Figure 2:**
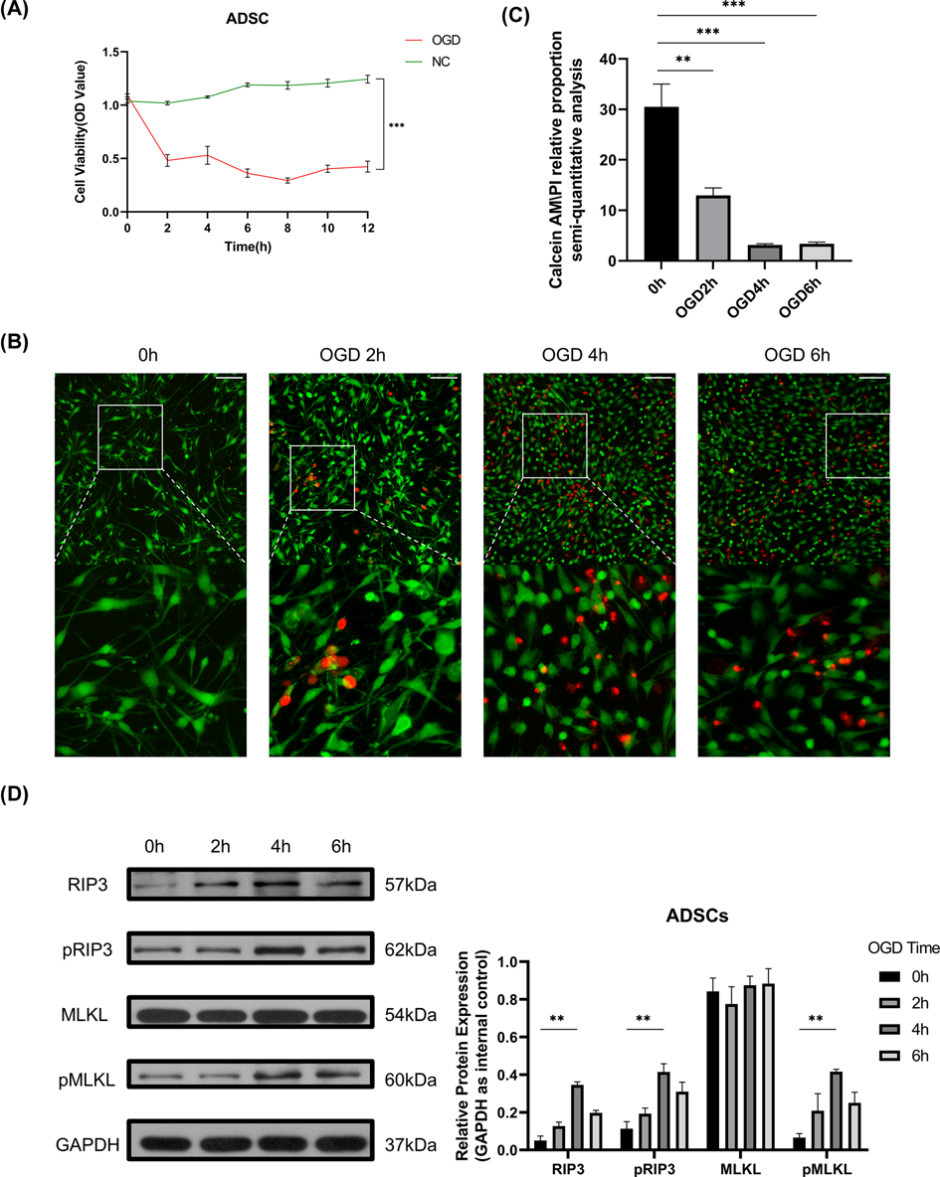
Changes in ADSCs under OGD treatment at different times (**A**) Cell viability curve measured by CCK-8 kit. ADSCs under NC or OGD model from 0 to 12 h, and significant difference persisted from 2 to 12 h. (**B**) ADSCs under OGD conditions from 0 to 6 h; calcein AM emitted green, representing living cells, and propidium iodide emitted red, representing dead cells with impaired cell membranes. Scale bars = 100 µm. (**C**) Proportion of living and dead cells calculated by ImageJ. ADSCs under the OGD model at 2/4/6 h had a lower living cell ratio than those at the beginning (12.96 ± 1.47%, 3.15 ± 0.24%, 3.37 ± 0.34% *vs* 30.52 ± 4.49%, respectively). (**D**) (Left) The protein levels of RIP3, pRIP3, and MLKL, and pMLKL at 0/2/4/6 h; (Right) RIP3, pRIP3, and pMLKL at 4 h had higher expression than at 0 h (10.94 ± 5.01-fold, 4.76 ± 1.73-fold, and 7.63 ± 1.95-fold, respectively). **P<0.01, ***P<0.001.

A growing body of studies has established an appropriate OGD model for various cell types; however, the effect of extreme hypoxia and a glucose-free environment on ADSCs remains unclear. To confirm the most suitable length of OGD time, we used the CCK-8 assay (Figure 2A), and the curve showed a statistical decrease in cell viability in the OGD group from 2 h to the endpoint compared with the normal control group, which indicated that a lack of glucose and oxygen suppressed cell proliferation efficiently and effectively. The cell viability at time points from 4 to 12 h showed no significant difference. Given that it took 2–3 weeks for angiogenesis to occur after fat transplantation, we did not set the conditions of reoxygenation and reglucose for follow-up studies. The appropriate OGD treatment time for ADSC culture *in vitro* remains unclear, so we focused on the changes in ADSCs at different times in the OGD environment. Since the extent of inhibition of cell activity reached its peak at 4 h, we further performed calcein AM/PI double staining on cells under OGD for up to 6 h. The live/dead proportion calculated by ImageJ following calcein AM/PI double staining in the three groups decreased as time passed from beginning to 4 h after OGD. Nevertheless, no significant change was observed between the 4- and 6-h groups (Figure 2B,C). The results indicated that the ratio of dead cells at 4 h was higher than that at the previous time points but not significantly different from that at 6 h. In summary, we chose 4 h as the follow-up OGD processing time. Subsequent Western blotting was performed to detect RIP3/pRIP3/MLKL/pMLKL, the essential proteins in the classic necroptosis pathway. As expected, the expression levels of RIP3, pRIP3, and pMLKL 4 h after OGD were higher than those in the control group, while the MLKL expression levels among the four groups showed no significant change (Figure 2D). The immunofluorescence results were basically consistent with the immunoblot results (Supplementary Figure S1). Given the results above, a 4-h OGD duration was used in subsequent experiments, and necroptosis probably occurred simultaneously.

### Inhibiting RIP3 ameliorated necroptosis in ADSCs

**Figure 3:**
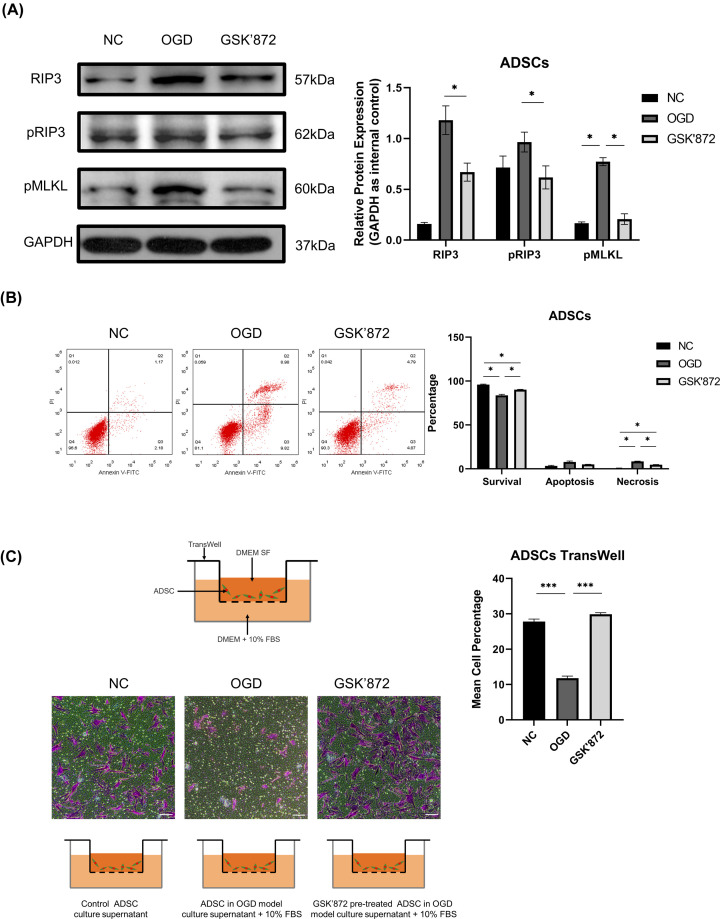
Inhibition of RIP3 and its effect on cell survival and migration (**A**) (Left) The expression of RIP3, pRIP3, and pMLKL was determined by Western blotting. (Right) GSK′872-ADSCs showed lower expression levels of RIP3, pRIP3, and pMLKL than OGD-ADSCs (0.57 ± 0.02-fold, 0.63 ± 0.06-fold, and 0.27 ± 0.08-fold, respectively). (**B**) The percentage of cells within the NC, OGD, and GSK′872 groups in the lower left (survival) quadrant was 95.93 ± 0.62%, 83.67 ± 1.32%, 90.17 ± 0.41%, in the lower right (apoptosis) quadrant was 3.23 ± 0.73%, 7.81 ± 1.13%, 5.04 ± 0.18%, and in the upper right (necrosis) quadrant was 0.59 ± 0.30%, 8.44 ± 0.33%, 4.75 ± 0.22%, respectively. The cell survival rate of the OGD group was lower than that of the NC group and GSK′872 group, and that of the GSK′872 group was lower than that of the NC group. The cell necrosis rate between groups demonstrated the opposite trend. In addition, no significant difference regarding the apoptosis rate was observed among these three groups. (**C**) The percentages of Crystal Violet-stained cells in the NC, OGD, and GSK′872 groups were 27.8 ± 0.72%, 11.8 ± 0.62%, and 30.0 ± 0.45%, respectively. A significant increase in the cell migration rate was observed in NC-ADSCs and GSK′872-ADSCs compared with OGD-ADSCs. Scale bars = 100 µm. *P<0.05, ***P<0.001.

The growth curve revealed that cells treated with GSK′872 at concentrations of 10.0, 5.0, and 2.5 µM exhibited suppressed proliferation, and GSK′843 indicated the same trend (Supplementary Figure S2); thus, we selected a concentration of 1 µM for GSK′872 and GSK′843 pretreatment in the following experiments. We explored whether the relative expression of necroptosis-associated proteins was elevated 4 h after OGD treatment. Western blotting assays indicated that RIP3/pRIP3/pMLKL expression in the two inhibitor groups was down-regulated compared with that in the OGD group but was still higher than that in the NC group (Figure 3A and Supplementary Figure S3). Hence, GSK′872 was chosen as the inhibitor in subsequent experiments, and ADSCs were separated into three groups: NC, OGD, and GSK′872. Annexin V/PI double staining showed similar results. ADSCs treated with GSK′872 under OGD for 4 h had more cells negative for both probes than the OGD group but fewer cells negative for both probes than the NC group. The corresponding rate of cells positive to both probes demonstrated an opposite trend between the three groups (Figure 3B).

### Cell migration

Transwell assays were carried out to test whether the OGD model affected the migration ability of ADSCs. Semiquantitative analysis showed that the number of Crystal Violet-stained ADSCs in the GSK′872 group was higher than that in the OGD group, and no significant difference was observed between the OGD group and the NC group (Figure 3C). This analysis suggested that the OGD process suppressed the cell migration ability of ADSCs and that inhibition of RIP3 reversed this effect.

### Adipogenesis

**Figure 4:**
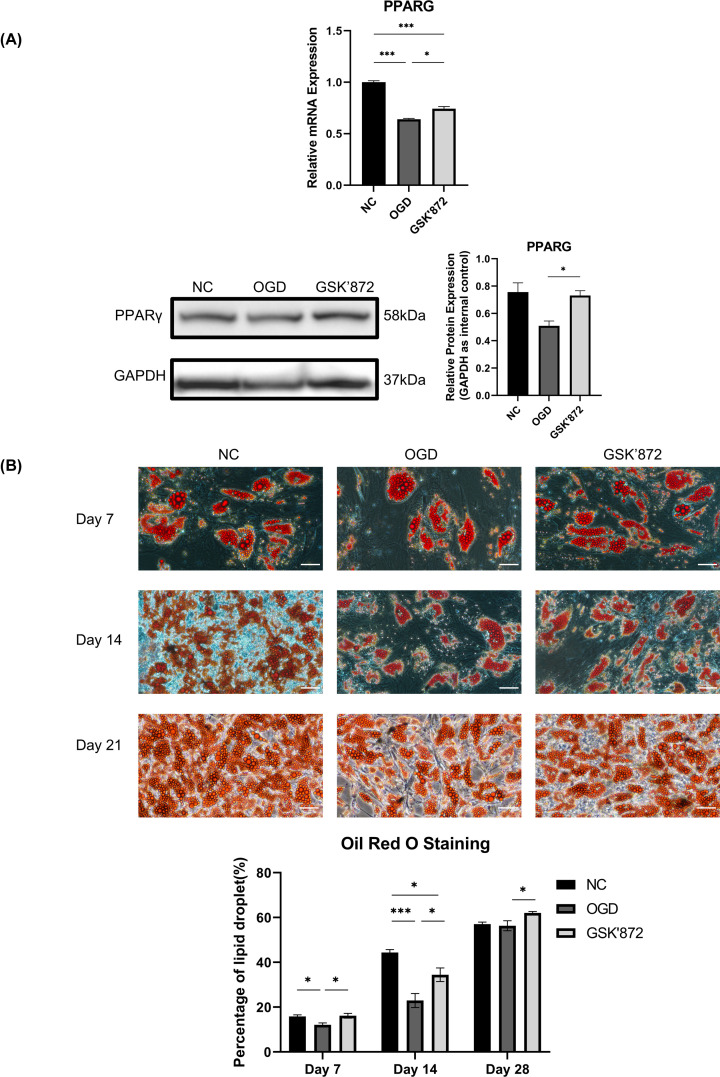
Change in adipogenesis (**A**) (Top) Relative mRNA expression levels in the three groups. *PPARγ* NC *vs* OGD *vs* GSK′872 = 1.00 ± 0.02 *vs* 0.64 ± 0.01 *vs* 0.74 ± 0.02. (Bottom left) The expression of PPARγ was determined by Western blotting. (Bottom right) GSK′872-ADSCs showed a higher expression level of PPARγ than OGD-ADSCs (1.44 ± 0.07-fold). (**B**) (Top) Oil Red O staining on days 7, 14, and 21 of adipogenic differentiation of the three groups. (Bottom) The percentages of lipid droplets on days 7, 14, and 21 in the three groups (NC-ADSCs *vs* OGD-ADSCs *vs* GSK′872-ADSCs) were 15.84 ± 0.71% *vs* 12.10 ± 0.90% *vs* 16.16 ± 1.01%, 44.39 ± 1.30% *vs* 22.94 ± 3.13% *vs* 34.46 ± 3.04%, 57.05 ± 0.90% *vs* 56.32 ± 2.22% *vs* 62.03 ± 0.69%, respectively. Scale bars = 50 μm. *P<0.05, ***P<0.001.

Quantitative RT-PCR and Western blotting for related adipogenic markers were conducted. The results showed significantly higher PPARγ expression at both the mRNA and protein levels in the GSK′872 group than in the OGD group (Figure 4A). Furthermore, the quantitative analysis of Oil Red O staining after adipogenesis induction indicated that ADSCs pretreated with GSK′872 demonstrated more adipogenic activity following OGD for 4 hdays 7 and 14 than those in the OGD group (Figure 4B).

### Vascularization

**Figure 5:**
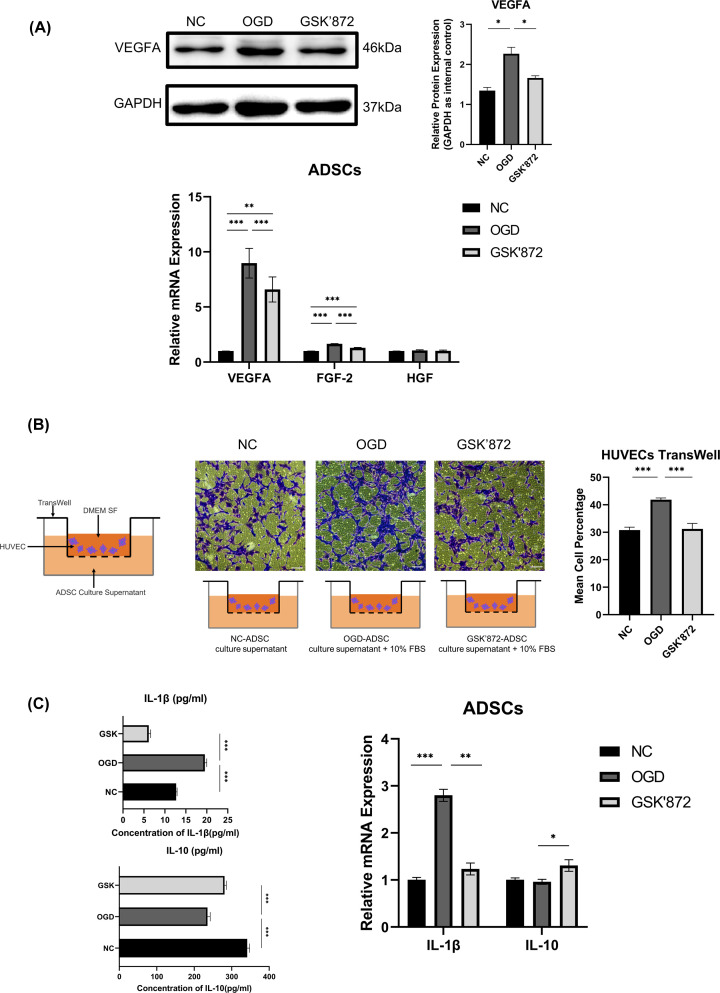
Changes in angiogenesis and inflammatory regulation (**A**) (Top left) VEGFA expression was determined by Western blotting. (Top right) OGD-ADSCs showed higher expression levels of VEGFA than NC-ADSCs and OGD-ADSCs (2.48 ± 0.29-fold and 1.54 ± 0.06-fold, respectively). (Bottom) Relative mRNA expression levels in the three groups. *VEGFA* NC *vs* OGD *vs* GSK′872 = 1.00 ± 0.01 *vs* 8.98 ± 1.35 *vs* 6.59 ± 1.14; *FGF-2* NC *vs* OGD *vs* GSK′872 = 1.00 ± 0.01 *vs* 1.65 ± 0.33 *vs* 1.29 ± 0.04; *HGF* NC *vs* OGD *vs* GSK′872 = 1.00 ± 0.01 *vs* 1.05 ± 0.07 *vs* 1.02 ± 0.07. (**B**) The percentages of Crystal Violet-stained cells in the NC, OGD, and GSK′872 groups were 30.79 ± 1.07%, 41.85 ± 0.62%, and 31.24 ± 2.00%, respectively. A significant increase in the cell migration rate was observed in OGD-HUVECs compared with NC-HUVECs and GSK′872-HUVECs. Scale bars = 100 µm. (**C**) (Left) Cytokine secretion level quantified by ELISA. The protein concentrations of IL-1β and IL-10 in the conditioned media of the three groups (NC *vs* OGD *vs* GSK′872) were 12.69 ± 0.24 *vs* 19.55 ± 0.44 *vs* 6.16 ± 0.44 pg/ml and 341.5 ± 6.4 *vs* 235.5 ± 7.1 *vs* 281.0 ± 4.8 pg/ml, respectively. (Right) Relative mRNA expression levels in the three groups. *IL-1β* NC *vs* OGD *vs* GSK′872 = 1.00 ± 0.05 *vs* 2.80 ± 0.13 *vs* 1.23 ± 0.13; *IL-10* NC *vs* OGD *vs* GSK′872 = 1.00 ± 0.04 *vs* 0.96 ± 0.05 *vs* 1.31 ± 0.12. *P<0.05, **P<0.01, ***P<0.001.

To confirm the ability of ADSCs to promote neovascularization stability, mRNA expression analysis of related genes was performed. An apparent up-regulation of VEGFA and fibroblast growth factor 2 (FGF2) occurred in OGD-ADSCs, while no significant difference was observed for the pattern of HGF. In addition, immunoblotting results showed the same trend of VEGFA among NC-ADSCs, OGD-ADSCs, and GSK′872-ADSCs (Figure 5A). To further validate whether ADSCs recruited more endothelial cells through secretion of cytokines, HUVECs seeded in a chamber were cocultured in a Transwell assay with supernatant from ADSCs at the bottom. Semiquantitative analysis showed that HUVECs cocultured with ADSC supernatant from the OGD group appeared more abundant on the Transwell membrane than those from the GSK′872 group and NC group. There was no significant difference between the latter two groups (Figure 5B). These results suggest that ADSCs can promote the migration and chemotaxis of endothelial cells through their paracrine function under OGD conditions.

### Inflammatory regulation

Gene expression analysis revealed that inhibiting RIP3 altered the mRNA expression levels of IL-1β and IL-10. A significant up-regulation of IL-1β was observed in OGD-ADSCs relative to GSK′872-ADSCs and was associated with a substantial down-regulation of IL-10. Overall, these results point to an alteration in the immunomodulatory features of OGD-ADSCs relative to GSK′872-ADSCs, with a shift toward proinflammatory behavior.

ELISA analysis of the cell lysates collected from the three groups revealed significantly higher concentrations of IL-1β in OGD-ADSCs than in GSK′872-ADSCs. In contrast, significantly lower concentrations of IL-10 were observed in GSK′872-ADSCs than in OGD-ADSCs (Figure 5C).

## Discussion

AFT technology is widely used in cosmetic and reconstructive surgery with continuous development. However, the instability of graft retention limits the further development, popularization, and application of this technology [21–23]. The theoretical basis of AFT mainly includes graft survival theory and graft replacement theory [24]. Yoshimura, one of the supporters of graft replacement theory, presented that the reconstruction of recipient capacity mainly benefits from the replacement of adipose stem/progenitor cells in the ischemic necrosis area [8,25]. In addition, he first proposed CAL with the stromal vascular fraction as an auxiliary ingredient, and scholars have successfully improved fat retention by mixing ADSCs into fat grafts, whereas controversies regarding CAL still exist [11,26–30]. The central area of the graft was in an extreme state of ischemia and hypoxia, the oxygen partial pressure was only approximately 15 mmHg, and neovascularization required up to 2–3 weeks [8]. Reducing the impact of extreme environments and maintaining the biological activity of ADSCs have become the key to improving the efficiency of CAL. To fit the environment of the necrotic area of the graft, we adopted the OGD model for research on ADSCs *in vitro*.

Degterev et al. first reported that specific cells undergo necroptosis in an OGD environment, which is a partially regulated necrosis-like death mode activated by the inhibition of caspase-8 [31]. In tumor necrosis factor receptor 1 (TNFR1) signaling, TNFα binds with TNFR1, inducing phosphorylation of RIP1 and downstream activities. When caspase 8 was inhibited, phosphorylated RIP1 recruited phosphorylated RIP3 to form necrotic bodies, and RIP3 induced the phosphorylation of MLKL, which oligomerized and migrated to the membrane, resulting in cell membrane disintegration, cell necrosis, and release of a large number of damage-related molecular patterns (DAMPs) [32]. Through the blot results of ADSCs after OGD treatment for 4 h, the necroptosis classical pathway proteins RIP3, pRIP3, and pMLKL were significantly higher than those in the control group, suggesting that necroptosis may be closely related to the changes in ADSCs in the OGD environment. RIP1 was not necessary for necroptosis; for example, TLR could directly activate RIP3, inducing necroptosis [33,34]. Therefore, we chose RIP3 as the intervention point for the subsequent experiment.

With the development of graft replacement theory, recent studies have demonstrated that ADSCs promote the migration of endothelial cells, maintain the stability of neovascularization [35–37] and increase the proliferation of new adipocytes [35]. In addition, ADSCs inhibited the inflammatory response in the graft’s nonperipheral area and reduced the degree of necrosis by secreting inflammatory regulators [36,37]. The effects above indirectly suggested the possible mechanism by which ADSCs promote the survival of fat grafts. As the contribution of ADSCs to graft retention may be underlied in migration, adipogenesis, angiogenesis, and inflammatory regulation, we used GSK′872, an effective and specific inhibitor of RIP3, to modulate these activities by inhibiting the kinase.

Flow cytometry showed that inhibition of RIP3 increased the survival rate of ADSCs with a decrease in RIP3, pRIP3, and pMLKL at the same time, which suggested that necroptosis inhibition might contribute to improving the live cell ratio [18]. Previous studies indicated that a mildly hypoxic environment promoted proliferation and migration, while anoxia induced the opposite trend [38,39]. The Transwell assay where fewer cells migrated to the membrane in the OGD group indirectly proved the effect of OGD on cell migration. The increase in cell death and suppressed migration ability in the central area of the graft, which are the two main mechanisms of decreasing the number of ADSCs in the adverse microenvironment, might be related to necroptosis. It could be concluded that the inhibition of RIP3 can reduce the cell mortality of ADSCs and retain their migration ability in the OGD environment.

ADSCs have the ability to undergo adipogenic differentiation, which is mainly regulated by PPARγ. PPARγ mediates the change in the epigenetic transition state of precursor adipocytes and maintains the gene expression of mature adipocytes [35,40–42]. The expression of PPARγ in the GSK′872 group was higher than that in the OGD group, which was consistent with the semiquantitative results of Oil Red O staining in adipogenic differentiation, suggesting that GSK′872 might protect the adipogenic potential of ADSCs by up-regulating PPARγ. To date, no evidence has been presented to prove the connection between necroptosis and adipogenesis, which indicated another underlying pathway beneath the positive interrelation of GSK′872 and PPARγ. A recent study demonstrated that GSK′872 regulated PPARγ in macrophages, possibly through the ROS-caspase 1 pathway [43]. As caspase-1 mediated cleavage and impaired the transcriptional activity of PPARγ, a higher concentration of caspase-1-dependent IL-1β in the OGD group from our ELISA results further indicated the possibility of enhanced PPARγ induced by inhibition of RIP3. PPARG further modulated the downstream end-stage adipogenic genes as a vital transcript factor, resulting in smaller droplet formation within ADSCs under OGD than ADSCs pretreated with GSK′872, consistent with the Oil Red O analysis [35,40,42,44,45].

VEGFA plays a crucial role in angiogenesis, which involves a multistep process including regulating vascular permeability, disintegrating the vessel wall, degrading the basement membrane, increasing the migration and invasion of extracellular matrix, and enhancing the proliferation of endothelial cells. It should be noted that excessive VEGFA will destroy the intracellular barrier, increase the leakage of endothelial cells, cause edema and activate the inflammatory pathway [46,47]. Hypoxia has been proven to be a crucial factor that might contribute to the higher expression of VEGFA [48–51] so that the mRNA and protein levels of VEGFA in OGD cells were higher than those in NCs. Interestingly, recent studies have suggested that RIP3 could act as a regulatory element of specific pathways to regulate VEGFA expression levels [47,52], which might counteract the enhanced VEGFA levels due to the inhibition of RIP3 and thereby induce a decrease in VEGFA expression in the GSK′872 group. FGF2 promotes the migration, proliferation, and tube formation of HUVECs [53]. Our results showed that GSK′872 reduced FGF2 expression in ADSCs in the OGD environment, possibly through necroptotic inhibition [54]. In conclusion, OGD caused VEGFA and FGF2 overexpression to affect angiogenesis and vascular stability, consistent with the HUVEC migration assay. GSK′872 reduced VEGFA and FGF2 overexpression by inhibiting RIP3 and increasing neovascularization stability.

IL-1β is an effective activator of dendritic cell subsets and a regulator of T-cell differentiation and function [55]; in contrast, IL-10 is the most crucial cytokine to inhibit the proinflammatory response and limit the overimmune response of various autoimmune diseases [56]. Through the qPCR and ELISA results, we found that OGD improved the secretion of IL-1β. RIP3 can increase IL-1β synthesis through multiple pathways, and the disintegration of the cell membrane caused by necroptosis further aggravates the release of IL-1β [57–61]. This explained why the application of GSK′872 reduced the IL-1β concentration. Interestingly, the IL-1β concentration in the GSK′872 group was even lower than that in the NC group. Our study simultaneously showed a higher level of IL-10 in the GSK′872 group, which indicated that the inhibition of necroptosis might increase the expression of IL-10. A previous study obtained similar evidence through the RIP3-knockout macrophage model [62]. Together, these results indicate that the inhibition of RIP3 modifies the inflammation regulating competency of ADSCs under OGD conditions by adjusting the secretion of the cytokines IL-1β and IL-10.

## Conclusion

In conclusion, appropriate OGD culture conditions *in vitro* imitated the possible environment of ADSCs *in vivo*. Through GSK′872 intervention in necroptosis, ADSCs enhanced resistance to an extremely hypoxic glucose-free state, improved cell viability, and modified the competency of adipogenesis, angiogenesis, and inflammation regulation. This put forward a more promising hypothesis for improving the efficiency of CAL and promoting fat retention.

## Supplementary Material

Supplementary Figures S1-S3

## Data Availability

All supporting data are included within the main article and its supplementary files. These data can be accessed and obtained by contacting the corresponding authors through email.

## Abbreviations

ADSC: adipose-derived stem cell
AFT: autologous fat transplantation
CAL: cell-assisted lipotransfer
CCK-8: cell counting kit-8
DAPI: 4′,6-diamidino-2-phenylindole
DMEM: Dulbecco’s modified Eagle’s medium
FGF2: fibroblast growth factor 2
GAPDH: glyceraldehyde 3-phosphate dehydrogenase
HUVEC: human umbilical vein endothelial cell
IL: interleukin
MLKL: mixed lineage kinase domain-like pseudokinase
MSCM: mesenchymal stem cell medium
OGD: oxygen-glucose deprivation
PBS: phosphate-buffered saline
PI: propidium iodide
pMLKL: phosphorylated MLKL
pRIP3: phosphorylated RIP3
PPARγ: peroxisome proliferator-activated receptor γ
qPCR: quantitative polymerase chain reaction
RIP: receptor-interacting protein kinase
VEGFA: vascular endothelial growth factor-A
