# Supplementary figures and images for: Inhibition of RIP3 increased ADSC viability under OGD and modified the competency of adipogenesis, angiogenesis, and inflammation regulation
Source: Biosci Rep. 2022 Mar 29;42(3):BSR20212808. doi: 10.1042/BSR20212808 (PMC8965819; doi:10.1042/BSR20212808)

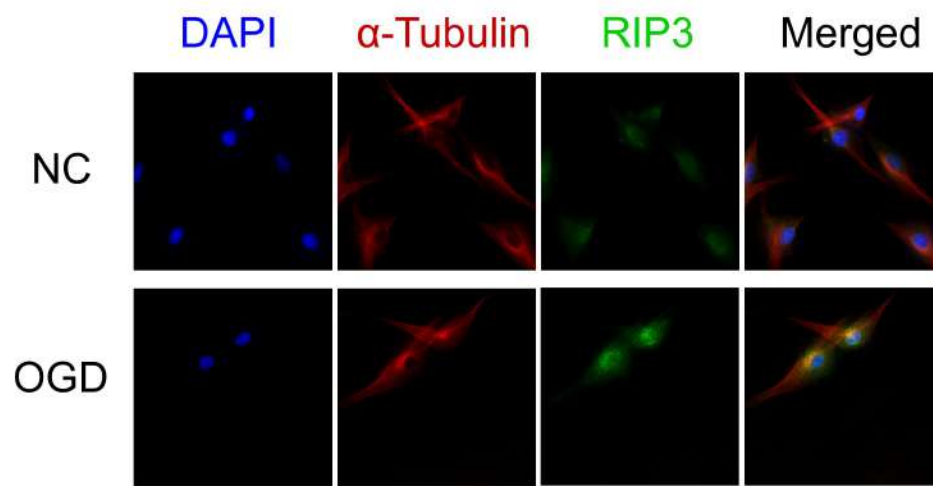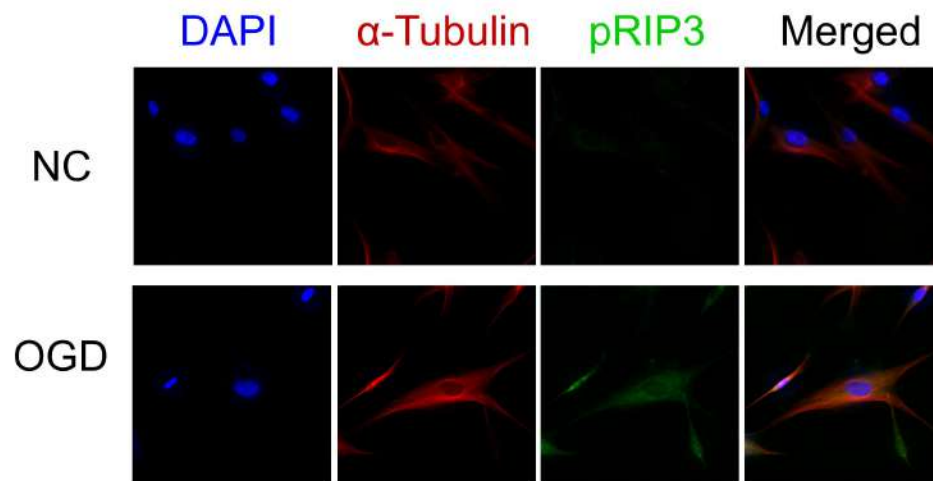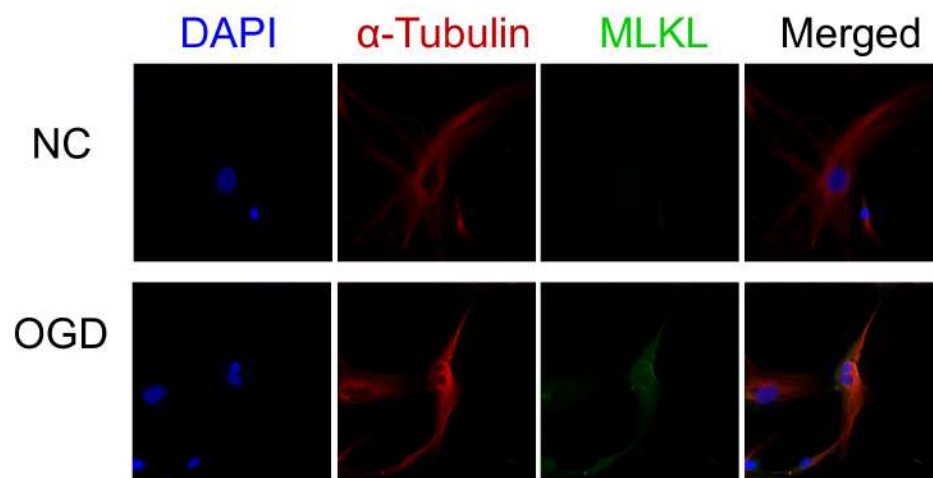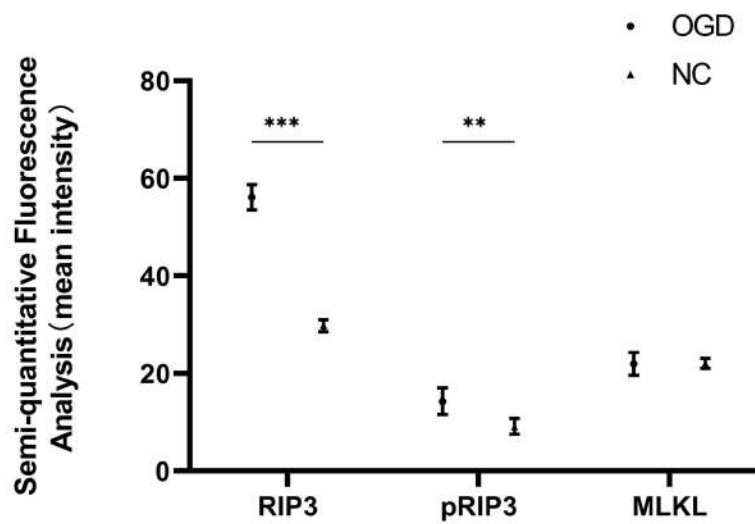

## ADSCs pretreated with GSK'872

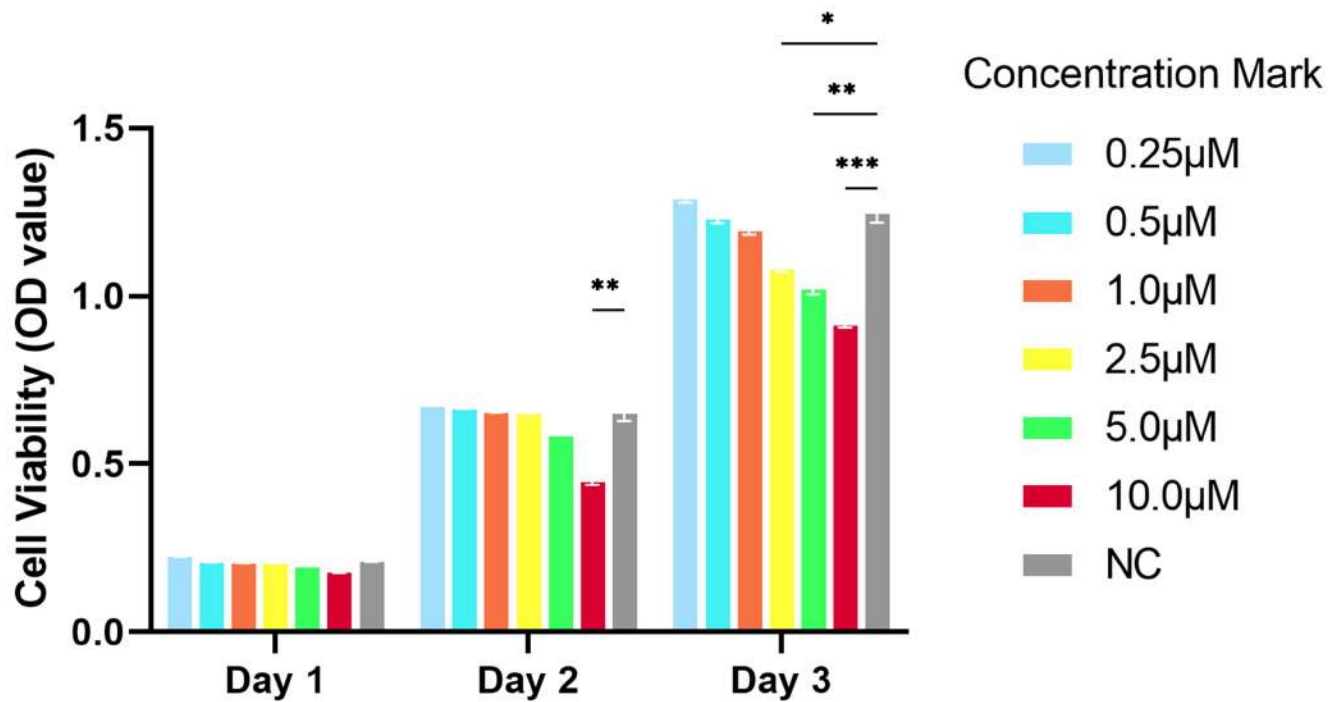

## ADSCs pretreated with GSK'843

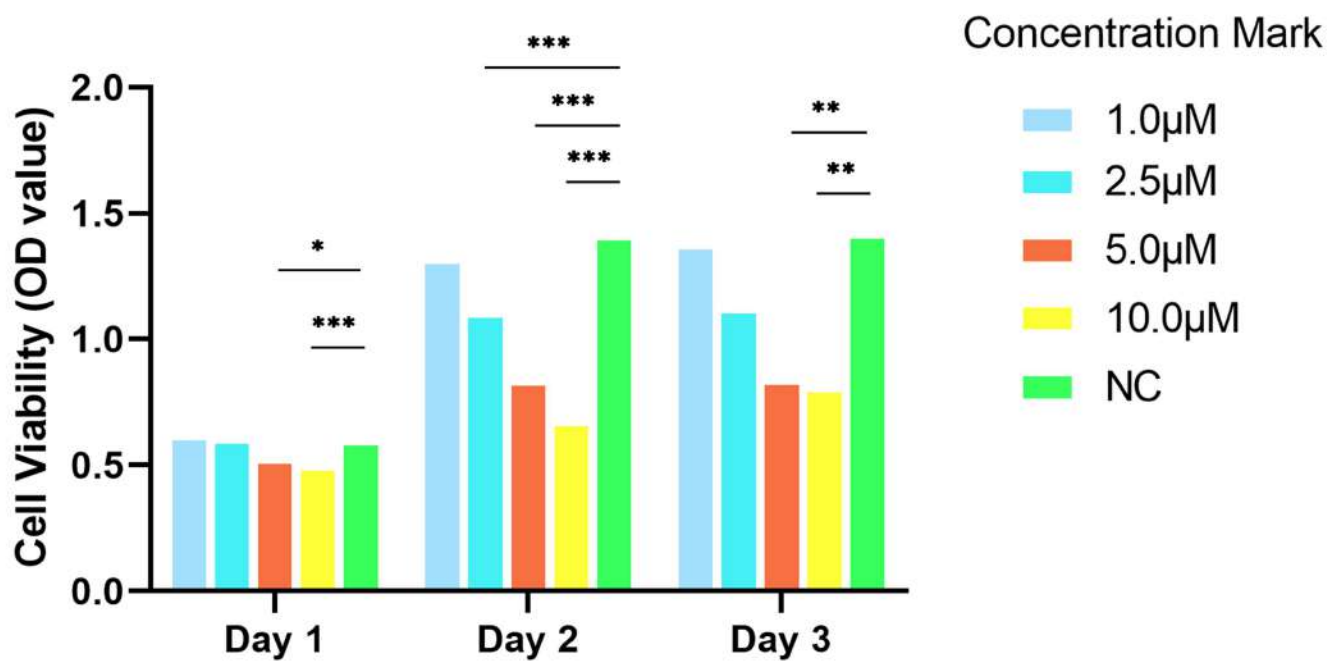

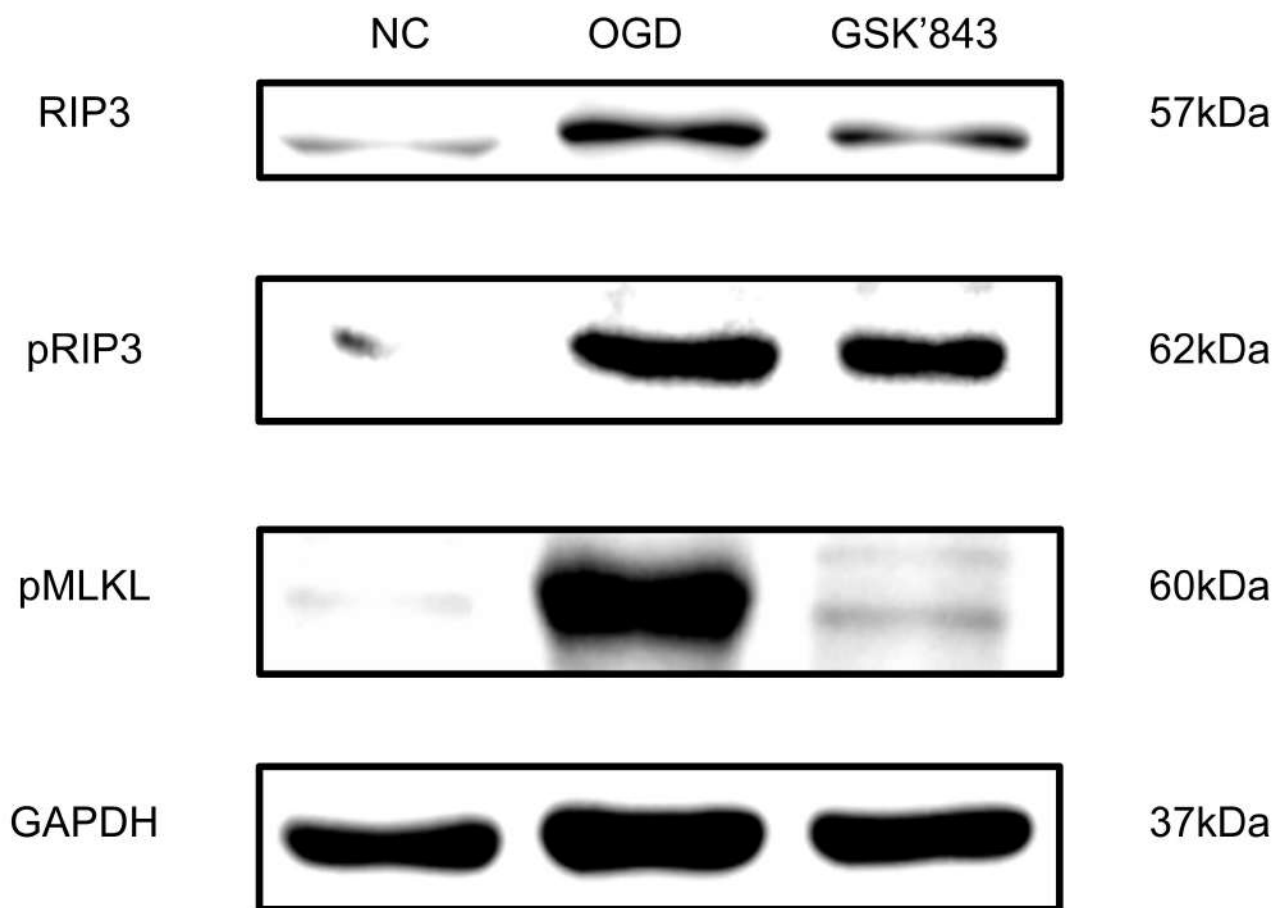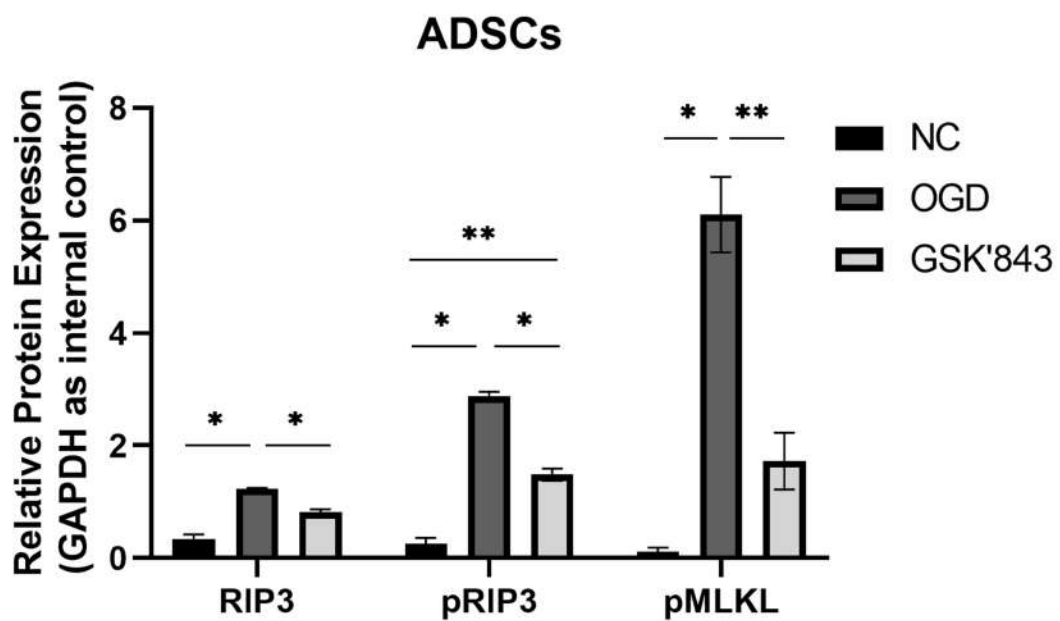

Supplement: Supplementary Figures S1-S3 [file BSR-2021-2808_supp.pdf]
